# Isolation of Three Novel Rat and Mouse Papillomaviruses and Their Genomic Characterization

**DOI:** 10.1371/journal.pone.0047164

**Published:** 2012-10-15

**Authors:** Eric Schulz, Marc Gottschling, Rainer G. Ulrich, Dania Richter, Eggert Stockfleth, Ingo Nindl

**Affiliations:** 1 Department of Dermatology, Venereology and Allergy, DKFZ-Charité Cooperation, Viral Skin Carcinogenesis Group, Charité, University Hospital, Berlin, Germany; 2 Department of Biology, Systematic Botany and Mycology, Ludwig-Maximilians-University Munich, GeoBio-Center, Munich, Germany; 3 Friedrich-Loeffler-Institut, Federal Research Institute for Animal Health, Institute for Novel and Emerging Infectious Diseases, Greifswald-Insel Riems, Germany; 4 Department of Parasitology, Institute for Pathology, Charité, University Hospital, Berlin, Germany; International Centre for Genetic Engineering and Biotechnology, Italy

## Abstract

Despite a growing knowledge about the biological diversity of papillomaviruses (PV), only little is known about non-human PV in general and about PV mice models in particular. We cloned and sequenced the complete genomes of two novel PV types from the Norway rat (*Rattus norvegicus*; RnPV2) and the wood mouse (*Apodemus sylvaticus*; AsPV1) as well as a novel variant of the recently described MmuPV1 (originally designated as MusPV) from a house mouse (*Mus musculus*; MmuPV1 variant). In addition, we conducted phylogenetic analyses using a systematically representative set of 79 PV types, including the novel sequences. As inferred from concatenated amino acid sequences of six proteins, MmuPV1 variant and AsPV1 nested within the Beta+Xi-PV super taxon as members of the Pi-PV. RnPV2 is a member of the Iota-PV that has a distant phylogenetic position from Pi-PV. The phylogenetic results support a complex scenario of PV diversification driven by different evolutionary forces including co-divergence with hosts and adaptive radiations to new environments. PV types particularly isolated from mice and rats are the basis for new animal models, which are valuable to study PV induced tumors and new treatment options.

## Introduction

Papillomaviruses (PV) are a diverse group of small, non-enveloped dsDNA viruses. The structurally conserved circular genome comprises approximately 8,000 bp and is organized in several, partially overlapping open reading frames (ORF). They include the major genes that are present in all PV, namely the early genes E1 and E2 as well as the late genes L1 and L2. Early proteins are involved in viral replication, life cycle, and regulation of viral gene expression, while the late proteins assemble the viral capsid. Besides the major ORF, additional early genes such as E4 and the viral oncogenes E6 and E7 are present in many PV, and E5 is encoded in, for example, genital Alpha-PV types.

PV infect epithelial tissues of the skin and mucosa at different regions in humans and various mammals, and most types causing epidermal proliferative lesions. Genital human PV (HPV) types are the etiological agent and the major risk factor of anogenital cancers (e.g., cervical cancer, vulvar cancer, penile cancer etc.) [1]. The viral oncogenes E6 and E7 are responsible for their ability to interfere with tumor suppressor genes, such as p53 and the retinoblastoma protein (pRB) family [2], [3]. By targeting p53 and pRB for proteasomal degradation, the viral oncogenes induce proliferation and transformation [4].

The molecular mechanisms of cutaneotropic HPV types (Beta- and Gamma-PV) are different compared to genital HPV. Nevertheless, cutaneotropic HPV types are very likely a cofactor in the early onset of cutaneous squamous cell carcinoma (SCC) [5]. The E6 proteins of cutaneotropic HPV types inhibit repair of UV-induced DNA-damage, induce genomic instability [6], [7], degrade the pro-apoptotic Bak protein [8]–[10], inhibit the proapoptocic function of p53 after DNA-damage [10], and generally inhibit apoptosis.

Traditionally, PV classification is inferred from the nucleotide sequence comparison of the L1 gene, defining nucleotide (nt) similarity thresholds for the delimitation of higher taxonomic units such as ‘type’, ‘species’, and ‘genus’ [11]. Moreover, phylogenetic analyses of concatenated genes produce well-supported molecular trees and identify four assemblages at high taxonomic level [12]–[14]. Based on those internal members most distantly related, we will refer to these supertaxa as Alpha+Omikron-PV, Beta+Xi-PV, Delta+Zeta-PV, and Lambda+Sigma-PV. Only a few isolated types cannot be assigned to one of these four large groups. The number of known non-human PV types has increased over the past few years resulting in 72 PV types, isolated from 36 mammalian and six sauropsid species [11]. However, knowledge about animal PV diversity is still poor by comparison to more than 150 known HPV types and only a few PV types have been described from more than 5,000 mammalian species, potential serving as hosts. Rodents belong to the better sampled mammal groups with respect to the presence of PV, and six types are presently known [15]–[22]. The majority of rodent-infecting PV types constitute the Pi-PV, while a single member each is known from the distantly related Iota- and Sigma-PV.

So far, different animal models have been used to examine PV induced tumors in natural occurring lesions of cattle, horse, dog, rabbit, and the multimammate rat [5]. These models are suboptimal because of the size of the animals and/or the lack of commercially available biochemical, biological, and immunological reagents. Thus, a non-transgenic mouse model, with naturally infectious PV types, is an ideal model system to examine PV induced diseases. Recently, a novel PV type MmuPV1 (originally designated MusPV) from a laboratory mouse (*Mus musculus*) has been isolated that naturally induce papilloma of the skin [23], [24]. Such reports are promising for the development of an effective animal model, and we here present and characterize the complete genomes of a new variant of MmuPV1 also isolated from a house mouse (MmuPV1 variant) as well as two novel PV types from a free ranging Norway rat (*Rattus norvegicus*, RnPV2), and a wood mouse (*Apodemus sylvaticus*, AsPV1). Rodent PV, isolated from rats and mice as classical laboratory animals, have great research importance, and we aim at contributing to an improved knowledge particularly of those organisms.

## Results

### Genome Organizations

**Figure 1:**
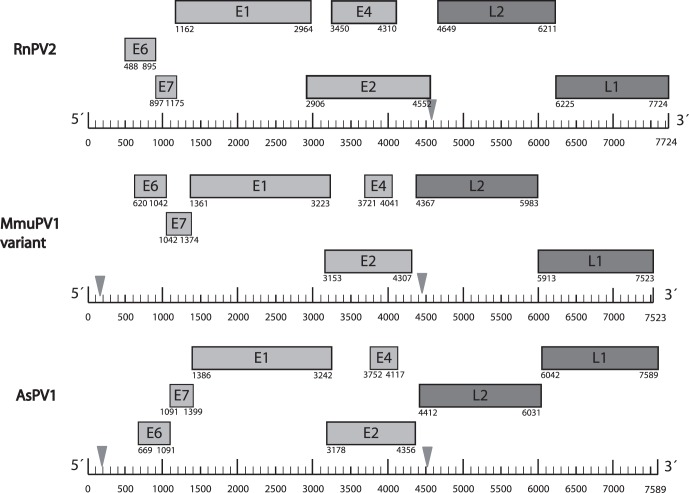
Genome organization of RnPV2, MmuPV1 variant, and AsPV1. Boxes indicating PV genes are drawn to scale and genes are drawn in three lines representing three putative open reading frames relative to nt position zero at the beginning of the upstream regulatory region. The polyadenylation sites are indicated with triangles.

PV associated benign or malignant tumors were not observed in the animals under investigation. We have isolated and cloned two novel PV types and one novel variant from three different rodent species. RnPV2 (HQ625441) was isolated from a rectal smear of *Rattus norvegicus*. RnPV2 DNA was additionally detected by PCR in hair follicles from the face of the same animal. Cutaneotropic AsPV1 (HQ625440) and MmuPV1 variant (HQ625439) were isolated from normal ear skin tissue of *Apodemus sylvaticus* and *Mus musculus*, respectively. The genomes of RnPV2, MmuPV1 variant, and AsPV1 consisted of 7,724 bp, 7,523 bp, and 7,589 bp, respectively (Fig. 1).

As inferred from protein prediction software programs and sequence evaluation in a comparative alignment, seven potential ORF coding for five early proteins (E1, E2, E4, E6 and E7) and two late proteins (L1 and L2) were identified, respectively (Fig. 1). The putative E4 ORF was characterized by proline codon-rich stretches, nested within the E2 ORF, and did not have a typical start codon (Table S1). Polyadenylation sites for early transcripts were identified in MmuPV1 variant at nt 4466 to 4471, in AsPV1 at nt 4511 to 4516, and RnPV2 at nt 4589 to 4594. Polyadenylation sites for late transcripts were only present in MmuPV1 variant and AsPV1 at nt positions 156 to 161 and 196 to 201, respectively (Fig. 1).

The deduced proteins of the new PV types harbored common amino acid (aa) motifs (Table S2), which were also present in other PV. They included two zinc finger motifs (C-X2-C-X29-C-X2-C) present in E6 and one zinc finger motif present in E7. Moreover, an ATP/GTP binding site (G-X4-GKS) was located within the SF3 helicase domain of E1. The E6 gene of MmuPV1 (both type and variant) had a potential pRB binding motif (L-X-C-X-E; aa positions 66 to 70), which was absent in other rodent PV. Another potential pRB binding motif was present in the N-terminal part of the RnPV2 E7 protein (aa positions 19 to 23). The E7 protein of MmuPV1 contained a potential PDZ binding domain (X-S/T-X-L/V; aa position 102 to 105) at the C-terminus, which was absent in other rodent PV types with the exception of MmPV. However, a stretch of seven aa between this putative motif and the end of the protein occurred in MmuPV1, while the PDZ binding motif of MmPV1 E7 was located at the ultimate C-terminus.

The L1 sequence of RnPV2 showed the highest nucleotide sequence similarity to MnPV1 (72% similarity), of AsPV1 to RnPV1 (75% similarity), and of MmuPV1 variant to MmuPV1 (99% similarity). A detailed comparison between the MmuPV1 type and the variant is shown in Table 1. All amino acids exchanges, which were not very likely altering the characteristics and charge of the protein, are indicated. Two insertions were present in the MmuPV1 variant of the E4 and the E2 gene resulting in one additional aa within each protein. Both insertions did not influence the following aa of the viral proteins (Table 1).

**Table 1:** Sequence comparison between MmuPV1 and the novel MmuPV1 variant.

Genes	E6	E7	E1	E2	E4	L2	L1
	**Position of ORF (including stop codon)**						
**MmuPV1 (7510 bp)**	610 to 1032	1032 to 1364	1351 to 3213	3143 to 4294	3711 to 4028	4354 to 5970	5900 to 7510
**MmuPV1 variant (7523 bp)**	620 to 1042	1042 to 1374	1361 to 3223	3153 to 4307	3721 to 4041	4367 to 5983	5913 to 7523
	**Lenght of ORF (including stop codon)**						
**MmuPV1**	423	333	1863	1152	318	1617	1611
**MmuPV1 variant**	423	333	1863	1155	321	1617	1611
	**Number of amino acids**						
**MmuPV1**	140	110	620	383	105	538	536
**MmuPV1 variant**	140	110	620	384	106	538	536
	**Sequence identities of nucleotides (amino acids)**						
	97% (98%)	98% (98%)	99% (100%)	99% (99%)	92% (93%)	99% (100%)	99% (99%)
	**Positions of amino acid insertion of MmuPV1 variant**						
				250 G	62 D		
	**Position of amino acid exchanges (MmuPV1/MmuPV1 variant)**						
	43 I/L	27 L/P	47 N/S	249 V/Y	48 P/Q	252 V/E	16 A/V
	44 Q/H	94 V/L	299 M/V	285/286 N/S	59 E/D	332 S/G	40 L/P
	139 L/S		501 M/V	286/287 S/I	60 Y/T		62 F/S
				358/359Y/H	83/84 Q/R		77 F/Y
					86/87 R/K		81 I/L
					95/96 T/A		
					96/97A/S		

ORF, open reading frame; bp, base pairs.

### Putative Transcription Factor Binding Sites (TFBS)

TFBS prediction identified a number of potential cis-regulatory elements in the upstream regulatory region (URR) of rodent PV. The URR sequences of rodent PV are comprised of a series of TFBS such as AP1, NF1, SP1, TEF1 and OCT1, which have been shown to be relevant in regulation of genital Alpha-HPV and cutaneous Beta-HPV gene expression (Fig. S1). Moreover, we identified potential TFBS, which have yet not been studied in relation to PV (Table S3). As in Alpha- and Beta-PV, the rodent PV URR was divided into a proximal and a distal enhancer element. The presence of cis-acting sequences in the proximal enhancer element was especially conserved, whereas their positions varied between different PV genera. In Pi- and Iota-PV, this region contained two E2 binding sites (BS), of which the proximal E2 BS was located directly upstream of a SP1 BS and downstream of a TFIID BS (TATA box binding protein). One potential E1 BS was always located between the two binding sites of E2. The proximal enhancer of the Sigma-PV EdPV1 was lacking the second E2 BS. In Pi-PV, the E2 BS in the proximal enhancer were located approximately 50 and 130 nt upstream of the transcription starting site. In contrast, the corresponding E2 BS of Iota-PV was located 65 and 115 nt upstream of the transcription start. Between the second E2 BS and the potential E1 BS of Iota-PV, a NF1 BS was situated 95 nt upstream of the transcription start. This motif was not present in Pi-PV.

The presence of cis-acting sequences in the distal enhancer element was only weakly conserved, even within the same PV genus. At least in most of the Pi-PV, a potential NF1 BS seemed to be conserved 265 to 260 nt upstream of the transcription start, whereas this motif was not present in Iota-PV. In Pi-PV, this NF1 BS was accompanied by one to two E2 BS, which were located upstream and downstream of the NF1 BS. They were always localized 210 and 290 to 310 nt upstream of the transcription start, respectively. In the distal enhancer element of Iota-PV, two E2 BS seemed to be conserved. They were located in close proximity to each other at 280 to 290 and 290 to 315 nt upstream of the transcription start. The presence of other potential TFBS seemed largely not to be conserved, even within the same PV genus and appeared to be randomly distributed.

### Phylogenetic Analysis

**Figure 2:**
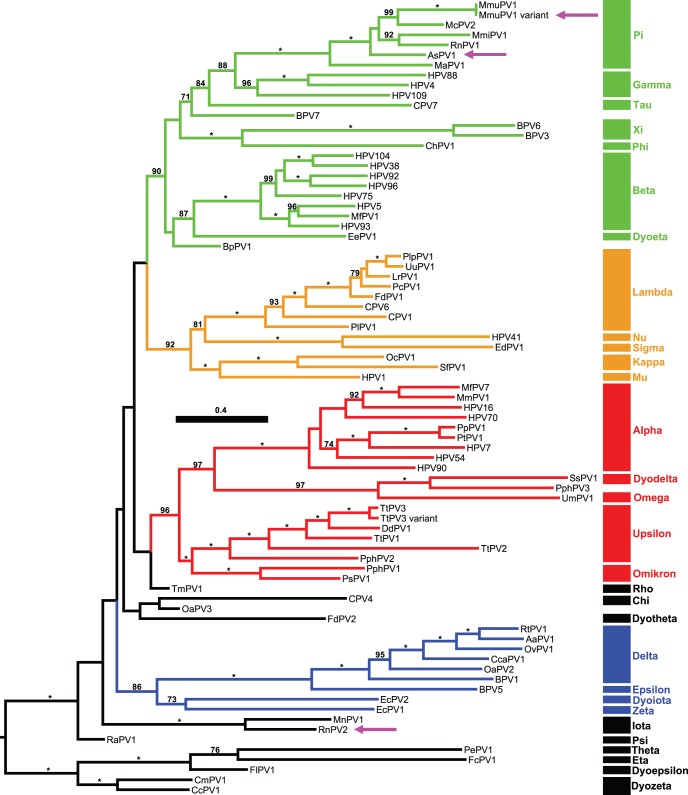
Maximum Likelihood (ML) tree of papillomaviruses (PV). ML tree comprising a representative set of 79 types including our two novel types (AsPV1, RnPV2) and one variant (MmuPV1 variant), as inferred from predicted E6, E7, E1, E2, L2, and L1 aa sequence analysis (3,720 aa positions, of which 69% were parsimony-informative). Generic PV clades [11] are indicated by Greek lettering. Supertaxa are colored red (Alpha+Omikron-PV), green (Beta+Xi-PV), blue (Delta+Zeta-PV), and ocher (Lamda+Mu-PV), respectively. Branch lengths are drawn to scale, with the scale bar indicating the number of nt substitutions per site. Numbers on branches are bootstrap support values under the ML criterion; values under 50 are not shown. The novel PV types and variant of our present study are highlighted by arrows. According to the common PV-nomenclature our novel variant of *Mus musculus* is referred to MmuPV1 variant.

The alignment consisted of 3,720 aa positions and 2,556 (69%) parsimony-informative sites (32.4 per terminal taxon; number of RAxML distinct alignment patterns: 3,555). In the best-scoring ML-tree based on concatenated E6–E7-E1–E2-L2–L1 aa sequences, mammalian PV were monophyletic (100 LBS) and segregated into the four supertaxa Alpha+Omikron-PV (96 LBS), Beta+Xi-PV (90 LBS), Delta+Zeta-PV (86 LBS), and Lambda+Mu-PV (92 LBS; Fig. 2). Rodent PV did not constitute a monophyletic group and were instead found at three different phylogenetic positions in the molecular tree: (i) Pi-PV (100 LBS, including the novel AsPV1 and MmuPV1 variant) within the Beta+Xi-PV, (ii) Sigma-PV (only EdPV1) within the Lambda+Mu-PV, and (iii) Iota-PV (100 LBS, including MnPV1 and the novel RnPV2) of uncertain phylogenetic position at the base of the tree. The closest relatives of Pi-PV were Gamma-PV (88 LBS) found on primates, while EdPV1 was the closest relative of HPV41 (100 LBS); the sister group of Iota-PV could not be determined reliably. With respect to the novel PV sequences, contradictory tree topologies between single genes as well as gene combinations were not observed (data not shown).

## Discussion

Despite the efforts that have been made to clone and to characterize novel PV types from different animal host species, knowledge about non-human PV diversity is generally sparse. The only two PV types naturally infecting common laboratory animals have been identified in the Norway rat (RnPV1) [22] and NMRI-Foxn1^nu^/Foxn1^nu^ (nude) mice (MusPV) [23], [24]. In our study, we present the complete genome sequences of two novel PV types and one variant, naturally infecting free ranging individuals of the Norway rat (RnPV2), the wood mouse (AsPV1), and the house mouse (MmuPV1 variant).

So far, several animal models with naturally infecting PV exist and have been used to examine molecular mechanisms of PV (including large domestic species such as rabbits, dogs, cattle, and horses). *Mastomys coucha* is the best examined laboratory animal model with naturally infecting PV types. MnPV1 and McPV2 are associated with benign and rarely malignant transformation of skin and mucosa, and this model is currently used to study PV-induced carcinogenesis [18], [25]. However, the complete genome of the host *Mastomys coucha* has not been sequenced yet and additionally immunological tools, i.e. antibodies are lacking. Thus, biological and immunological mechanisms of carcinogenesis are very difficult to examine. Recently, a novel PV type (MmuPV1) has been cloned from laboratory mice [23], [24]. MmuPV1 induces papilloma at the nose and mouth of nude mice NMRI-Foxn I^nu^/Foxn I^nu^, which are transmissible to other immunocompetent laboratory mice. Based on the high sequence similarity it is very likely that the novel MmuPV1 variant isolated in our study will have the same properties, which are warranted to examine in further studies.

Papillomavirus diversity results from different evolutionary mechanisms [14], [26]–[28] such as co-divergence with the hosts, lateral transfer (i.e., host switch), and adaptive radiation establishing new ecological niches. The existence of three, only distantly related rodent PV lineages (namely Iota-, Pi-, and Sigma-PV) rejects the hypothesis of co-divergence between the viruses and their hosts at a global scale [22], [28]. Even more strikingly, the two different PV types each isolated from *Rattus norvegicus* and *Mastomys coucha*, respectively, do not constitute monophyletic groups. However, a co-phylogenetic structure can be stated at least between Muridae [29] and Pi-PV, as far as the weakly resolved nodes in both viral and host trees allow for this observation. Based on robust phylogenetic inference determination and quantification of the different evolutionary forces that have led to PV diversity remain a major future task.

The viral tropism is rather conserved among the different PV lineages, although evolutionary shifts of this trait have taken place, for example, within the Alpha-PV [30]. The majority of types in this group are mucosotropic, with the exception of the cutaneotropic species Alpha-2 PV and Alpha-4 PV. Character polarity is not distinct among rodent PV, but it is noteworthy to state that each group, Iota- and Pi-PV, does include both cutaneotropic and mucosotropic types. Neither cutaneotropic nor mucosotropic types constitute monophyletic groups within Pi-PV (the lineage with the most rodent PV types known), which requires more than one evolutionary step in terms of this character trait to explain. We have isolated RnPV2 genome from a rectal smear and have detected RnPV2 DNA additionally in hair follicles from the face of the same individual. Thus, at present it is unknown whether lesions are induced by RnPV2, and the tropism is also unclear. Therefore studies examining the potential role of the new rodent PV during the development of skin or anogenital cancer are warranted.

The presence of TFBS such as AP1, NF1 [31], SP1 [32], TEF1 and OCT1 [33] have been shown to be relevant in regulation of Alpha-HPV gene expression. Potential binding sites of further cellular transcription factors within the PV URR have not been experimentally studied in relation to PV. We show here that the presence of potential TFBS, and their relative position to the transcriptional start site, is very similar in rodent PV belonging to the same genus, but different in rodent PV belonging to different genera. Genital Alpha- and cutaneous Beta-PV also exhibit conserved URR organizations among PV types belonging to the same genus. But the positions of specific TFBS within the URR are different comparing PV types from two different genera [34], [35]. Differences in URR organizations of genital Alpha- and cutaneous Beta-PV largely coincide with a different tissue tropism of the respective PV. This may reflect adaptations of the viral regulation to different environments. Pi- and Iota-PV also represent different PV genera with partially identical host range among rodents. The only data about tissue tropism of rodent PV are available of MnPV1 and McPV2 isolated from skin tumors and genital tumors of *Mastomys coucha* [18], [25]. From most rodent species, only one PV type has been isolated indicating the lack of knowledge about tissue tropism of EdPV, MmiPV, MaPV1, and RnPV1 [15], [20]–[22]. Therefore, it is not clear whether each of the different organizations of the URR in the respective rodent PV is reflected by a different tropism, which might be a general feature for all PV.

Putative E6 and E7 proteins of MmuPV1 variant and RnPV2 display aa motifs that have been shown to be responsible for transforming properties of high-risk HPV types. RnPV2 E7 has a potential pRB binding motif in its N-terminal domain that has been first described and exists in the E7 protein of high-risk HPV types [2]. E6 of MmuPV1 and MmuPV1 variant contains a potential pRB binding motif, which is usually present in E7 proteins. Moreover, E7 of both types contains a C-terminal PDZ binding motif that is normally a feature of E6 [3]. Interestingly, the PDZ binding motif in E7 of MmuPV locates at the C-terminus as MmPV1 E7 from Rhesus macaques, which has been shown to interact with cell polarity and cell motility [36]. Thus, functional studies will clarify these alternative binding motifs of MmuPV1 and the novel variant.

In our present study, we have isolated the whole genome of two novel wild living rodent PV types and a variant of a house mouse. Moreover, we have characterized the sequences of the novel PV types and have performed phylogenetic analysis. This information will be helpful to better understand the evolutionary processes and can serve as a basis to establish powerful animal models with laboratory mice or rats. The animal models will be valuable to study molecular mechanisms of PV induced lesions, their interaction with the immune system and novel therapeutic treatments.

## Materials and Methods

### Ethics Statement

Animal ethics obtained for the work were not required because the animals were either from a pest controller (Rattus norvegicus, Mus musculus) or cooperation partner from forestry institutions (Apodemus sylvaticus), which caught and sacrificed the animals during their official duties. The rodents were used with permission of the pest-control agencies or forestry agencies and institutions (Deutscher Schädlingsbekämpfer-Verband e.V., Landesverband Berlin/Brandenburg e.V., Berlin, Germany; Forstliche Versuchs- und Forschungsanstalt Baden-Württemberg, Freiburg, Germany). No experiments with live animals were performed. The rodents were killed by the third parties either by means of spring-loaded mouse traps or rodenticides. Trapping of the animals was conducted in the framework of monitoring activities for a hantavirus outbreak and was coordinated by the Friedrich-Loeffler-Institut, the Federal Research Institute for Animal Health. The responsible governmental bodies are the Senat von Berlin and the Regierungspräsidium Baden-Württemberg, Germany. The animals were handled according to the national and European legislation, namely the EU council directive 86/609/EEC for the protection of animals.

### DNA Isolation and Cloning

All samples (Table S1) were collected under sterile conditions. They included genital rectal smears of a Norway rat (*Rattus norvegicus*; female, adult, field sample: Berlin, Germany); 10–15 eyebrow hair bulbs from the same animal; normal skin from the ear of a house mouse (*Mus musculus*; female, adult, field sample: Berlin, Germany) and a wood mouse (*Apodemus sylvaticus*; female adult, field sample: Baden-Württemberg, Germany). Genomic DNA was isolated as previously described [37] and was stored at −20°C until further analysis.

Rolling Circle Amplification (RCA) was performed to amplify episomal PV DNA as described previously [38]. RCA products were used as templates to generate DNA fragments of approximately 450 bp by PCR with the degenerated FA-primers for a L1-fragment: [39], [40] and CP-primers for an E1-fragment: [41]. The DNA amplicons were separated by electrophoresis, purified, and sequenced. PCR products were cloned into the PCR-TOPO 2.1 vector (Invitrogen; Carlsbad, CA, USA), accordingly to manufacturer’s instructions. The L1 gene was used to design novel type-specific primers to amplify the complete genome. Primer sequences in 5′→3′ orientation used for the respective PV types were *RnPV2-F:* AAG GCA TTT GCA TTT GGA TC; *RnPV2-R:* CGT AAG GGG CGG GTA CTA TC; *AsPV1-F:* ATT GGT GTC TGT CTC TGC TT; *AsPV1-R:* CTG GCA AGT CCT ACT TTT GT; *MmuPV1-F:* TGG GTT TCT TAA AGG TCG TG; *MmuPV1-R:* ACG CCT GGG CAT TCT TAT T*G* *.* To amplify the whole genomic regions of the respective PV types, a final concentration of 0.5 µM primer pairs, 0.2 mM dNTPs, 0.5 mM MgCl_2_, 5 U LongAmpPol polymerase (NEB; Frankfurt/Main, Germany), and buffer containing 1.5 mM MgCl_2_ was used. After an initial denaturation step at 94°C for 2 min, 35 cycles of 94°C for 10 sec, 60°C for 30 sec, and 68°C for 6 min were carried out, and the PCR was completed by a final extension step at 68°C for 10 min. Resulting amplicons of approximately 7.5 kb were cloned into the PCR-TOPO 2.1 vector. The complete genomes were sequenced by a primer walking strategy (GLC Genomics; Berlin Germany). New PV-types were designated RnPV2, MmuPV1 variant, and AsPV1. All novel PV types are available upon request (IN).

### Protein Prediction

ORF were predicted using ‘ORFinder’ (http://www.ncbi.nlm.nih.gov/gorf/gorf.html). They were confirmed by manual alignment of nucleotide and protein sequences to homologous regions of most similar PV types using the ‘Se-Al’ program (v2.0a72; http://tree.bio.ed.ac.uk/software/seal/). Primary sequence analysis of the predicted proteins was performed with ‘ProtParam’ [42] and ‘Prosite’ (www.expasy.ch/prosite.html). TFBS were predicted with ‘Cister’ (http://zlab.bu.edu/~mfrith/cister.shtml) and ‘MATCH’ (www.generegulation.com/cgi-bin/pup/programs/match/bin/match.cgi), as previously described [38].

### Phylogenetic Analyses

The taxon sample for phylogenetic analyses was representative for the currently known PV diversity and comprised 79 complete sequences including the three novel PV sequences. An aa alignment of the proteins E6–E7-E1–E2-L2-L1 was concatenated using ‘MAFFT’ v6.523 [43]; http://align.bmr.kyushu-u.ac.jp/mafft/software/). The data matrix is available upon request (IN). Phylogenetic analyses were run using distinct models/data partitions (i.e., six for each gene), with individual per partition branch length optimization. To explore possible topological incongruence, concatenated (all six proteins as well as E1–E2 and L2-L1 gene combinations) and separate analyses of single genes were performed. Maximum likelihood-based analyses were conducted using the PTHREADS version of ‘RAxML’ VII (http://www.phylo.org/portal/Home.do) and applying the rtREV+G substitution model, as previously described [14].

### Note

The following GenBank/EMBL/DDBJ accession numbers are reported in this paper: RnPV2 (HQ625441), MmuPV1 variant (HQ625439) and AsPV1 (HQ625440).

## Supporting Information

Figure S1 **Potential binding partners of the upstream regulatory regions from rodent PV types.** Predictions of several transcription factor binding sites (TFBS) (e.g. binding sites of viral E1 and E2, TFBS), which have been shown to be involved in transcriptional regulation of PV are drawn to scale. Asterisks indicate that the respective binding site is degenerated at one nt position.
(EPS)

Table S1 **Primary sequence analysis of the putative genes from MmuPV1 variant, AsPV1, and RnPV2, respectively.**
(DOC)

Table S2 **Potential functional elements of the putative proteins from the novel PV types.**
(DOC)

Table S3 **Predicted transcription factor binding sites and other regulatory elements in the rodent PV genomes.**
(DOC)
