# Supplementary material for: Isolation of Three Novel Rat and Mouse Papillomaviruses and Their Genomic Characterization
Source: PLoS One. 2012 Oct 15;7(10):e47164. doi: 10.1371/journal.pone.0047164 (PMC3471917; doi:10.1371/journal.pone.0047164)
Supplement: Table S1 — Primary sequence analysis of the putative genes from MmuPV1 variant, AsPV1, and RnPV2, respectively. (DOC) [file pone.0047164.s002.doc]

**Supplementary material**

**Table S1. Primary sequence analysis of the putative genes from MmuPV1 variant**, AsPV1, and RnPV2, respectively.

| Gene | E6 | E7 | E1 | E2 | E4 | L2 | L1 |
| --- | --- | --- | --- | --- | --- | --- | --- |
| MmuPV1 variant | | | | | | | |
| No. of nucleotides [bp] | 423 | 333 | 1863 | 975 | 321 | 1617 | 1530 |
| No. of amino acids | 140 | 110 | 620 | 324 | 106 | 538 | 509 |
| molecular weight [kDa] | 15.78 | 12.03 | 70.12 | 43.56 | 11.72 | 57.34 | 60.59 |
| isoelectric point [pH] | 8.86 | 4.06 | 5.75 | 6.90 | 10.94 | 4.62 | 7.54 |
| AsPV1 | | | | | | | |
| No. of nucleotides [bp] | 423 | 309 | 1857 | 1179 | 366 | 1620 | 1548 |
| No. of amino acids | 140 | 102 | 618 | 392 | 121 | 539 | 515 |
| molecular weight [kDa] | 16.06 | 11.35 | 69.96 | 44.16 | 13.16 | 57.74 | 58.44 |
| isoelectric point [pH] | 8.92 | 4.67 | 5.86 | 8.98 | 7.11 | 4.63 | 8.42 |
| RnPV2 | | | | | | | |
| No. of nucleotides [bp] | 408 | 279 | 1803 | 1647 | 861 | 1563 | 1500 |
| No. of amino acids | 135 | 92 | 600 | 548 | 286 | 520 | 499 |
| molecular weight [kDa] | 15.53 | 10.27 | 68.40 | 59.85 | 31.86 | 56.14 | 56.80 |
| isoelectric point [pH] | 9.42 | 4.59 | 5.91 | 9.62 | 8.84 | 5.76 | 8.28 |

No., number.
