# Supplementary material for: Isolation of Three Novel Rat and Mouse Papillomaviruses and Their Genomic Characterization
Source: PLoS One. 2012 Oct 15;7(10):e47164. doi: 10.1371/journal.pone.0047164 (PMC3471917; doi:10.1371/journal.pone.0047164)
Supplement: Table S2 — Potential functional elements of the putative proteins from the novel PV types. (DOC) [file pone.0047164.s003.doc]

**Supplementary material**

**Table S2. Potential functional elements of the putative proteins from the novel PV types.**

| Name | aa positions (within protein) | Motif |
| --- | --- | --- |
| **MmuPV1 variant** | | |
| Zincfinger motif | 27…63 (E6) | C-X2-C-X29-C-X2-C |
| Zincfinger motif | 100…136 (E6) | C-X2-C-X29-C-X2-C |
| pRB binding motif | 66…70 (E6) | X-L-X-C-X-E |
| Zincfinger motif | 63…100 (E7) | C-X2-C-X29-C-X2-C |
| PDZ binding motif | 102…105 (E7) | X-S/T-X-L/V |
| ATP/GTP-binding site, motif A (P-loop) | 446...453 (E1, ATP-dependent helicase domain: 420…570) | G-X4-GKS |
| **AsPV1** | | |
| Zincfinger motif | 27…63 (E6) | C-X2-C-X29-C-X2-C |
| Zincfinger motif | 100…136 (E6) | C-X2-C-X29-C-X2-C |
| Zincfinger motif | 54…90 (E7) | C-X2-C-X29-C-X2-C |
| ATP/GTP-binding site, motif A (P-loop) | 446 …453 (E1, ATP-dependent helicase domain: 420 … 570) | G-X4-GKS |
| **RnPV2** | | |
| Zincfinger motif | 23…59 (E6) | C-X2-C-X29-C-X2-C |
| Zincfinger motif | 96…132 (E6) | C-X2-C-X29-C-X2-C |
| Zincfinger motif | 49…85 (E7) | C-X2-C-X29-C-X2-C |
| pRB binding motif | 19…23 (E7) | L-X-C-X-E |
| ATP/GTP-binding site, motif A (P-loop) | 428 …435 (E1, ATP-dependent helicase domain: 402 …552) | G-X4-GKS |

(aa, amino acids).
