# Supplementary material for: Isolation of Three Novel Rat and Mouse Papillomaviruses and Their Genomic Characterization
Source: PLoS One. 2012 Oct 15;7(10):e47164. doi: 10.1371/journal.pone.0047164 (PMC3471917; doi:10.1371/journal.pone.0047164)
Supplement: Table S3 — Predicted transcription factor binding sites and other regulatory elements in the rodent PV genomes. (DOC) [file pone.0047164.s004.doc]

**Supplementary material**

**Table S3. Predicted transcription factor binding sites and other regulatory elements in the rodent PV genomes**.

| Factor name | Function | nt positions (5’🡪3’) | Motif (5’🡪3’) |
| --- | --- | --- | --- |
| **AsPV1** | | | |
| Tef | TFBS | 13 ... 24 | ctcattcctgta |
| OCT1 | TFBS | 23…37 | tataatgtgaattga |
| OCT1 | TFBS | 57...70 | ctacatgtaatgtg |
| OCT1 | TFBS | 62…76 | tgtaatgtgaattgt |
| OCT1 | TFBS | 91…98 | aattgcac |
| FOXJ2 | TFBS | 130 ... 143 | gacacaatattgta |
| ER | TFBS | 133 ... 151 | acaatattgtatgaccggt |
| AP-1 | TFBS | 153 ... 163 | gctgaccggct |
| MyoD | TFBS | 168 ... 179 | ggacatgtgcta |
| AP-1 | TFBS | 185 ... 195 | actgacctatg |
| poly A-signal | mRNA processing | 196 …201 | aataaa |
| AP-1 | TFBS | 200 ... 210 | aatgactaacg |
| Tef | TFBS | 220 ... 231 | cacaagcctcgc |
| AREB6 | TFBS | 241 ... 253 | ccgcacctgtgt |
| AP-1 | TFBS | 260 ... 270 | agtgccagagt |
| E2 | TFBS | 338…353 | ggaccgtttacggttt |
| Pax-6 | TFBS | 340 ... 360 | accgtttacggtttaatccaa |
| NF-1 | TFBS | 360 ... 377 | agttggcagacgtctaga |
| NF-1 | TFBS | 380 ... 397 | ctttggcaaagtgtttag |
| Ap1 | TFBS | 477 …483 | tgagtaa |
| NF1 | TFBS | 512 ….517 | ttggc |
| E2 | TFBS | 532...547 | cgaacgggaacggtac |
| E1 | viral origin | 563 …593 | aagcagtgattgttgccaacaactatcatac |
| Sp1 | TFBS | 610 …614 | ggtgg |
| E2 | TFBS | 613…629 | ggagcgttaacggtcg |
| Ap1 | TFBS | 629…635 | tgtataa |
| TATA | promoter element | 630 ... 644 | gtataaataagcctg |
| poly A-signal | mRNA processing | 4,511 …4,516 | aataaa |
| **MmuPV1 variant** | | | |
| v-Myb | TFBS | 14 ... 23 | actaactgaa |
| AP-1 | TFBS | 62 …68 | tgtataa |
| poly-A signal | mRNA processing | 156 ... 161 | aataaa |
| AP-1 | TFBS | 160 ... 170 | aatgactaatg |
| AREB6 | TFBS | 202 ... 213 | tcgcacctgggc |
| NF-1 | TFBS | 239 ... 268 | aaacagtctctgttggctgtgtgctctct |
| NF1 | TFBS | 251 …255 | ttggc |
| E2 | TFBS | 297 ... 312 | ggaccgttttcggtcg |
| NF1 | TFBS | 344 …348 | tggc |
| AP-1 | TFBS | 360 ... 370 | aatgactaact |
| E2 | TFBS | 407 ... 423 | gtaccgttttcggtcg |
| TATA | Promoter element | 424 ... 438 | gtaaaaaaggcgcca |
| AP-1 | TFBS | 446 ... 456 | catgattcaga |
| YY1 | TFBS | 460 …466 | ccattgt |
| E2* | TFBS | 491 …502 | ttctggaacggt |
| E1 | viral origin | 516… 546 | tttgagctgatggttggcaacaattatttcc |
| COMP1 | TFBS | 521 ... 544 | gctgatggttggcaacaattattt |
| NF1 | TFBS | 529 …533 | tggc |
| Sox-5 | TFBS | 532 ... 541 | gcaacaatta |
| Sp1 | TFBS | 562 …566 | ggtgg |
| E2 | TFBS | 565 ... 580 | ggagcgggaacggtcg |
| TATA | promoter element | 580 ... 594 | gcatataagtatcag |
| poly A-signal | mRNA processing | 4,466 ... 4,471 | aataaa |
| **RnPV2** | | | |
| YY1 | TFBS | 15 …21 | acatcgt |
| E2 | TFBS | 34 ... 49 | gcaccgcctcgggttt |
| Sp1 | TFBS | 50 ... 62 | tgggggtggagct |
| NF1 | TFBS | 75 …79 | ttggc |
| MyoD | TFBS | 78 ... 89 | gcacaggtgggg |
| NF-1 | TFBS | 88 ... 105 | ggttggcagaagggctcg |
| Ap1 | TFBS | 134 …140 | ttactca |
| E2 | TFBS | 167 ... 182 | gcaccttcctcggttt |
| Tef | TFBS | 168 ... 179 | caccttcctcgg |
| E2 | TFBS | 194 ... 209 | acaccgaattcggtac |
| FOXD3 | TFBS | 214 ... 225 | ttttttttcttt |
| NF-1 | TFBS | 249 ... 266 | ttctggctcggttcctgt |
| c-Ets-1(p54) | TFBS | 267 ... 276 | ccaggacgtg |
| Ap1 | TFBS | 287 …293 | ttattca |
| TATA | promoter element | 331 ... 345 | ccacgaattttatcc |
| Hand1:E47 | TFBS | 346 ... 361 | ccttcggtctgggata |
| E2 | TFBS | 366 ... 381 | aaaccgatatgggtgt |
| NF-1 | TFBS | 385 ... 402 | tgttggcaacaatcctct |
| E1 | viral origin | 392 …423 | aacaatcctctctctatactttccgatctcga |
| Tef | TFBS | 408 ... 419 | tactttccgatc |
| E2 | TFBS | 422 ... 437 | gaaccggtaccggtca |
| TATA | promoter element | 443 …449 | tatttaa |
| Barbie Box | TFBS | 449 ... 463 | agcaaaaggtggtgg |
| Poly A signal | mRNA processing | 4,589 …4,594 | aataaa |

TFBS, transcription factor binding site.
